# Validation of the BOADICEA model for predicting the likelihood of carrying pathogenic variants in eight breast and ovarian cancer susceptibility genes

**DOI:** 10.1038/s41598-023-35755-8

**Published:** 2023-05-26

**Authors:** Nanna Bæk Møller, Desirée Sofie Boonen, Elisabeth Simone Feldner, Qin Hao, Martin Larsen, Anne-Vibeke Lænkholm, Åke Borg, Anders Kvist, Therese Törngren, Uffe Birk Jensen, Susanne Eriksen Boonen, Mads Thomassen, Thorkild Terkelsen

**Affiliations:** 1 Department of Clinical Genetics, Aarhus University Hospital, Brendstrupgårdsvej 21, 8200 Aarhus N, Denmark; 2 Department of Clinical Genetics, Odense University Hospital, J. B. Winsløws Vej 4, 5000 Odense, Denmark; 3 Department of Surgical Pathology, Zealand University Hospital, Roskilde, Denmark; 4 Division of Oncology, Department of Clinical Sciences Lund, Lund University, Lund, Sweden

**Keywords:** Cancer genetics, Genetic testing, Breast cancer, Gynaecological cancer, Cancer epidemiology

## Abstract

BOADICEA is a comprehensive risk prediction model for breast and/or ovarian cancer (BC/OC) and for carrying pathogenic variants (PVs) in cancer susceptibility genes. In addition to *BRCA1* and *BRCA2*, BOADICEA version 6 includes *PALB2*, *CHEK2*, *ATM*, *BARD1*, *RAD51C* and *RAD51D*. To validate its predictions for these genes, we conducted a retrospective study including 2033 individuals counselled at clinical genetics departments in Denmark. All counselees underwent comprehensive genetic testing by next generation sequencing on suspicion of hereditary susceptibility to BC/OC. Likelihoods of PVs were predicted from information about diagnosis, family history and tumour pathology. Calibration was examined using the observed-to-expected ratio (O/E) and discrimination using the area under the receiver operating characteristics curve (AUC). The O/E was 1.11 (95% CI 0.97–1.26) for all genes combined. At sub-categories of predicted likelihood, the model performed well with limited misestimation at the extremes of predicted likelihood. Discrimination was acceptable with an AUC of 0.70 (95% CI 0.66–0.74), although discrimination was better for *BRCA1* and *BRCA2* than for the other genes in the model. This suggests that BOADICEA remains a valid decision-making aid for determining which individuals to offer comprehensive genetic testing for hereditary susceptibility to BC/OC despite suboptimal calibration for individual genes in this population.

## Introduction

Around two percent of the general population carry a pathogenic variant (PV) in a susceptibility gene for breast cancer and/or ovarian cancer (BC/OC)^1,2^. Carriers of PVs in BC/OC susceptibility genes benefit from targeted interventions depending on their personal risk profile, such as increased surveillance or risk-reducing surgery. As comprehensive genetic testing remains costly, however, guidance is required to identify which individuals to offer screening for PVs. Over the years, multiple algorithms have been developed to predict the likelihood of carrying a PV in *BRCA1* or *BRCA2*^3^. The Breast and Ovarian Analysis of Disease Incidence and Carrier Estimation Algorithm (BOADICEA) is a widely used breast cancer risk assessment tool in European genetics clinics^4^, and its validity to predict the likelihood of PVs in *BRCA1* and *BRCA2* has been demonstrated in several large-scale studies^5,6^. In its latest implementations as part of the CanRisk suite of BC/OC risk prediction models, the model has been expanded to include *PALB2*, *CHEK2*, *ATM*, *BARD1*, *RAD51C* and *RAD51D*^7,8^. This makes BOADICEA the most comprehensive model available to predict the likelihood of PVs in BC/OC susceptibility genes. Yet its performance to predict PVs in genes other than *BRCA1* and *BRCA2* remains to be evaluated in an independent cohort. In this study, we validated its performance in more than two thousand individuals examined for germline PVs in BC/OC susceptibility genes at clinical genetics departments in Denmark.

## Methods

### Ethics declaration

The study was conducted in accordance with the Declaration of Helsinki. All methods involving human participants and data were performed following the relevant guidelines and regulations. The research ethics committee system of Denmark consisted of local institutional review boards and the National Committee on Health Research Ethics of Denmark. The national committee had the authority to waive the requirement for informed consent in studies that included comprehensive genetic information of the participants; however, ethics approval was deemed unnecessary by the committee with reference to the legislation for registry-based studies since the data had been generated in the clinical setting (ref. 66314). In Denmark, the requirement for consent from the participants and ethics approval was waived by law for registry-based studies. In accordance with the Danish data protection legislation, the study was registered with the Region of Southern Denmark (ref. 19/29487), and the Danish Patient Safety Authority granted permission to the study of medical records (ref. 3-3013-3247/1).

### Study population

The study population was identified in the registry of the clinical genetics laboratory at Odense University Hospital, which performed Next Generation Sequencing of a 64-gene panel during clinical testing for PVs in *BRCA1* and *BRCA2* in the period from 1 January 2013 to 31 December 2018. The patient samples originated from two geographic areas: Odense University Hospital in the Region of Southern Denmark (1.2 million inhabitants) and Aarhus University Hospital in the Central Denmark Region (1.3 million inhabitants). Genetic testing for BC/OC susceptibility was offered in public hospitals free of charge and guided by national referral criteria from the Danish Breast Cancer Cooperative Group to identify individuals in high-risk families. Genetic testing was preferentially offered to affected individuals; secondarily, to the nearest related relatives (Supplementary Table S1). The departments did not have to adhere to these criteria, however, so genetic testing could also be offered according to clinical decision. The inclusion criteria for the study were the availability of Next Generation Sequencing data meeting the sequencing quality criteria and an identified pedigree at the clinical genetics department of sample origin.

### Data sources

#### Pedigrees

During pre-test counselling, a genetics professional recorded a pedigree of the patient’s family history, which, as general rule, included at least three generations tracing back from the patient. The pedigree records included the source of information, vital status, date of birth, age, and cancer history of family members. Cancer diagnoses were validated in the medical records of family members wherever possible during the genetic work-up, which would result in a high validation rate for the cancer diagnoses^9^. For this study, each pedigree was collected into a pedigree data bank to include the three-generation family history of the patient: full siblings, parents, grandparents, aunts, and uncles. Cancer diagnoses were included in the data bank regardless of the source of information. The pedigree data bank contained the following records: id, sex, familial relations, year of birth, age, vital status, ages of breast cancer, bilateral breast cancer, ovarian cancer (including cancer in the ovaries, fallopian tube and primary peritoneal cancer), prostate cancer and pancreatic cancer, and breast cancer subtype by the expression pattern of estrogen receptor (ER) and human epidermal receptor 2 (HER2). Progesterone receptor (PR) expression was not routinely evaluated in Denmark. Instead progesterone receptor expression was assumed to correspond to the estrogen receptor expression^10^. Information about breast cancer expression of cytokeratin 14 and cytokeratin 5/6 was unavailable and therefore not included in the study. Male breast cancer was assumed to be unilateral and missing for subtype in accordance with the model.

#### Pathology information

By linkage to the personal identification number of the proband^11^, all available records were obtained from the Danish Pathology Data Bank^12^. This is a detailed registry of all specimen analyzed in pathology laboratories in Denmark. The first records in the registry span from 1960, but the registry is incomplete until 1997 when the registry became nationwide. The registry uses a Danish version of the Systemized Nomenclature of Medicine codes with officially updated versions available at its website (www.patobank.dk). All records in the registry must contain at least one topography code and morphology code. In addition, the records may contain codes for etiology, function, disease, and procedures, which denote additional information such as origin, hormone receptor status etc. A diagnosis of cancer was identified on the basis of a primary cancer of the topography of interest or a direct invasion or metastasis from the origin of interest. If the origin was unspecified for a direct invasion or metastasis, ovarian cancer was assumed in the case of a serous carcinoma and breast cancer in the case of an invasive ductal or lobular carcinoma. In situ cancer was excluded. Information about receptor expression was deemed to be valid if the date of the specimen was within one year of the diagnosis with breast cancer.

#### Pathogenic variants

Sample preparation for Next Generation Sequencing was performed using 3 µg DNA by Agilent’s SureSelectXT Reagent kit and custom designed adaptors. Target enrichment was performed using a custom 64-gene SureSelextXT panel designed at Lund University Hospital. The panel is designed to aim even coverage and includes introns for *BRCA1* and *BRCA2*. Sequencing was performed using 2 × 75 bp sequencing of 16 samples in an Illumina NextSeq High Output flow cell. A minimum coverage of 20 × in ≥ 95% of the aimed regions was required to meet the sequencing quality criteria. The typical mean coverage was > 500 ×. Data alignment and small variant calling were processed using Illumina DRAGEN Bio-IT Platform (Illumina, San Diego, CA, U.S.). Sequencing data was aligned to human genome reference GRCh37. VCF files for small variants were imported to VarSeq (Golden Helix, Bozeman, MT, U.S.) for annotation. Variants were annotated using RefSeq transcripts, and filtered by DRAGEN quality, coverage, and population frequency. Only the variants within coding region + 20 bp padding were evaluated. Copy-number variants (CNVs) were called by VarSeq CNV caller (Golden Helix, Bozeman, MT, U.S.). Whole exon was used as the target region for CNV calling. The clinical significance of both small variants and CNVs were evaluated manually. Gene specific ACMG guidelines from ClinGen including the expert panel ENIGMA (https://clinicalgenome.org/) were applied for variant classification. Class 4 (likely pathogenic) and class 5 (pathogenic) variants were considered to be PVs in the evaluation of BOADICEA, which included PVs in *BRCA1*, *BRCA2*, *PALB2*, *CHEK2*, *ATM*, *BARD1*, *RAD51C* and *RAD51D*. For *BRCA1* and *BRCA2* all variant types were included, also missense PVs. For the remaining genes, only predicted truncating variants were included in accordance with the BOADICEA model^8^. No actions were taken to blind the assessment.

### Data analysis

The sample size for the study was determined by the available data. Predicted carrier likelihoods of PVs in *BRCA1*, *BRCA2*, *PALB2*, *CHEK2*, *ATM*, *BARD1*, *RAD51C* and *RAD51D* were obtained from BOADICEA v6.1.0 by analysis of the pedigrees on the CanRisk server (https://canrisk.org/boadicea/)^8^. The pedigrees included the proband and their family members up to 2nd degree relatives, including year of birth, age of death, cancer history and breast cancer subtype as described in the previous sections. Predictors were only included up to the known age of the individual at the time of the genetic test result. Polygenic risk scores and multifactorial risk factors were not included, nor cytokeratin 14 and cytokeratin 5/6. No actions were taken to blind assessment of the predictors. The BOADICEA default settings for PVs (mut_freq = UK) and the population-specific cancer incidence rates for Denmark were used (cancer_rates = Denmark). A sensitivity analysis was performed with all settings at their default values (mut_freq = UK, cancer_rates = UK). BOADICEA requires the year of birth, age and age of diagnosis for an individual to be included in the model. Data validation and imputation of missing data for these variables were performed as described in the supplement (see Supplementary Methods). The results for the whole study population after data imputations were compared with complete-case analysis of only individuals with complete information on the imputed variables.

The accuracy of the predicted likelihoods was investigated in terms of the calibration and discrimination of BOADICEA. The calibration of the model was evaluated by the observed-to-expected ratio (O/E), which compared the observed number of carrier of pathogenic variants (O) with the expected number of carriers of pathogenic variants (E). The expected number of PV carriers was determined by summation of the predicted likelihoods assuming that the sensitivity of genetic testing was 100%. To assess the impact of assuming reduced sensitivity of genetic testing, the predicted PV carrier likelihoods were multiplied by the current CanRisk default sensitivities for the BOADICEA model, assuming CNV analysis was performed for all genes, in which case the sensitivity of genetic testing was assumed to be reduced only due to unclassified missense PVs in *BRCA1* (sensitivity 89%) and *BRCA2* (sensitivity 96%)^8^. To examine the calibration of the model across the spectrum of PV carrier probabilities, the individuals were stratified into different categories of combined predicted likelihood of a PV in the genes evaluated^6^. The confidence interval for the O/E ratio was derived from the uncertainty of estimation for the observed number while assuming that the expected number was given without error^13^. The two-sided 95% confidence interval for the O/E ratio was hence calculated from the upper and lower bounds of the exact binomial confidence interval for the observed number^14^.

Calibration plots were constructed with the Stata module “pmcalplot” for the defined categories of predicted likelihood for all genes combined and for five equally sized quantiles of predicted likelihood for the individual genes. Statistical evidence of deviation from optimal calibration was evaluated by the Hosmer–Lemeshow Χ^2^ test using the predicted probabilities from BOADICEA as implemented in the Stata module “hl”.

The discriminative ability of the model was evaluated by the area under the receiver operating characteristics (ROC) curve (AUC), which compares the sensitivity and specificity of a model. The range of the AUC is between 0.5 (no apparent accuracy) and 1.0 (perfect accuracy) for a predictive model^15^. The AUC and its two-sided 95% confidence interval was estimated using DeLong’s method^16^. The sensitivity and specificity of the model were reported at potentially clinically relevant thresholds of the carrier probability from 0 to 20%.

The data analysis was performed in Stata v17.0 (StataCorp, USA).

## Results

The validation study included 2,033 individuals tested for PVs in BC/OC susceptibility genes during counselling at clinical genetics departments in Denmark (Table 1).

**Table 1:** Demographic data of the study cohort (N = 2033).

Age, mean (range)	54.1 (20–94)
Sex, female (%)	1863 (91.6)
Cancer* (%)	1621 (79.7)
Breast (%)	1334 (82.3)
Male (%)	26 (2.0)
Female (%)	1308 (98.0)
ER status known (%)	1127 (86.2)
ER− (%)	274 (24.3)
ER+ (%)	853 (75.7)
HER2 status known (%)	982 (75.0)
HER2− (%)	801 (81.6)
HER2+ (%)	181 (18.4)
Bilateral (%)	190 (14.5)
Ovarian (%)	301 (18.6)
Pancreatic (%)	18 (1.1)
Prostate (%)	32 (2.0)
Family history* (%)	1544 (76.0)

*Breast, ovarian, pancreatic or prostate cancer. *N* Total number of individuals, *ER* Estrogen receptor, *HER2* Human epidermal receptor 2.

### Calibration

**Figure 1:**
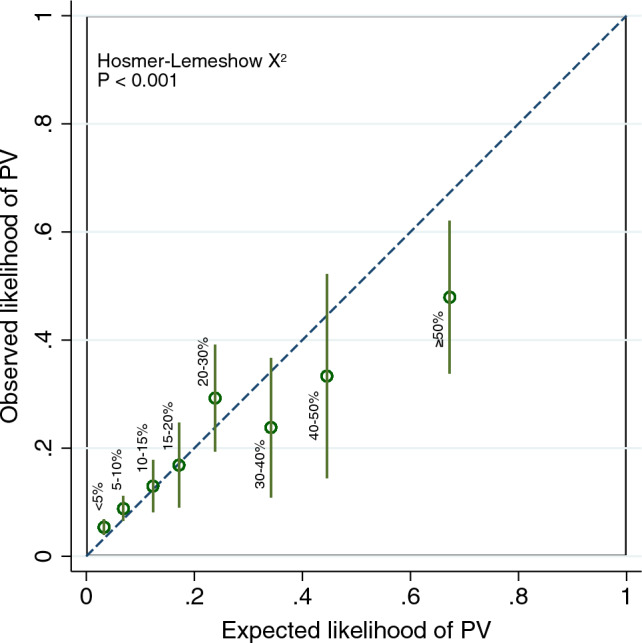
This calibration plot corresponds to Table 2. It shows the expected and observed likelihood of a PV according to the category of predicted carrier probability for *BRCA1*, *BRCA2*, *PALB2*, *CHEK2*, *ATM*, *BARD1*, *RAD51C* or *RAD51D*. The dashed line corresponds to a model with optimal calibration. The spikes (green) show the 95% CI for the observed likelihoods.

To evaluate the calibration of BOADICEA, we compared the observed number of carriers of PVs in the cohort with the expected number from the model. BOADICEA accurately predicted the combined likelihood of carrying PVs in the genes *BRCA1*, *BRCA2*, *PALB2*, *CHEK2*, *ATM*, *BARD1*, *RAD51C* and *RAD51D*. Thus, the ratio between the observed and expected PV carriers in all genes combined was 1.11 (95% CI 0.97–1.26). Using the default settings with UK cancer incidence rates instead of the population-specific incidence rates for Denmark yielded very similar results with an overall O/E of 1.07 (95% CI 0.94–1.22). The model remained largely well-calibrated at the different categories of predicted likelihood with moderate departures from unity at the lowest and highest of carrier probabilities as evident from the calibration plot (Fig. 1 and Table 2). This miscalibration was statistically significant (Hosmer–Lemeshow Χ^2^, P < 0.001).

**Table 2:** Calibration according to the predicted likelihood of carrying a PV in *BRCA1*, *BRCA2*, *PALB2*, *CHEK2*, *ATM*, *BARD1*, *RAD51C* or *RAD51D.*

CP (%)	N	O	E	O/E (95% CI)
< 5	985	53	32.4	1.64 (1.23–2.12)
5–10	578	51	39.8	1.28 (0.96–1.66)
10–15	185	24	22.8	1.05 (0.69–1.51)
15–20	89	15	15.3	0.98 (0.57–1.53)
20–30	82	24	19.6	1.23 (0.83–1.69)
30–40	42	10	14.4	0.70 (0.35–1.15)
40–50	24	8	10.7	0.75 (0.35–1.24)
≥ 50	48	23	32.3	0.71 (0.49–0.93)
All	2,033	208	187	1.11 (0.97–1.26)

*CP* Carrier probability for PVs (any gene), *N* Total number of individuals, *O* Observed carriers of PVs, *E* Expected carriers of PVs. Numbers for the individual genes can be found in the supplementary material (see Supplementary Table S2).

In the stratified analyses, the model underestimated the combined likelihood of PVs in *BRCA1* and *BRCA2* (O/E 1.38; 1.17–1.61) and overestimated this likelihood for the other genes in the model (O/E 0.78; 95% CI 0.60–0.99). In both sub-analyses, there was a trend towards underestimating the likelihood of PVs at increasing carrier probabilities (Supplementary Table S3); however, there was statistical evidence of miscalibration for *BRCA1*/*BRCA2* only (Hosmer–Lemeshow Χ^2^, P < 0.001) and not for the other genes in the model at these cut-offs for the predicted carrier probability (P = 0.13). Assessing calibration at quantiles of the predicted likelihood yielded similar results (Supplementary Fig. S1).

The model remained broadly well-calibrated regardless of the clinical data of the proband but was slightly better calibrated for individuals with a cancer diagnosis (O/E 1.06; 95% CI 0.92–1.22) than for unaffected individuals (O/E 1.48; 95% CI 1.02–2.07). The model overestimated the likelihood of carrying PVs for individuals with a personal or family history of bilateral breast cancer and, likewise, underestimated this likelihood for individuals with a personal or family history of ovarian cancer somewhat (Supplementary Table S4).

We also assessed the impact of assuming reduced sensitivity of genetic testing due to unclassified missense PVs in *BRCA1* and *BRCA2*. With these assumptions, the ratio between the observed and expected PV carriers was 1.16 (95% CI 1.01–1.32), indicating that the model will remain largely well-calibrated with improvements in classification of missense variants.

### Discrimination

The receiver-operating characteristics of the model indicated an acceptable overall discrimination between carriers and non-carriers of PVs with an AUC of 0.70 (95% CI 0.66–0.74).

**Figure 2:**
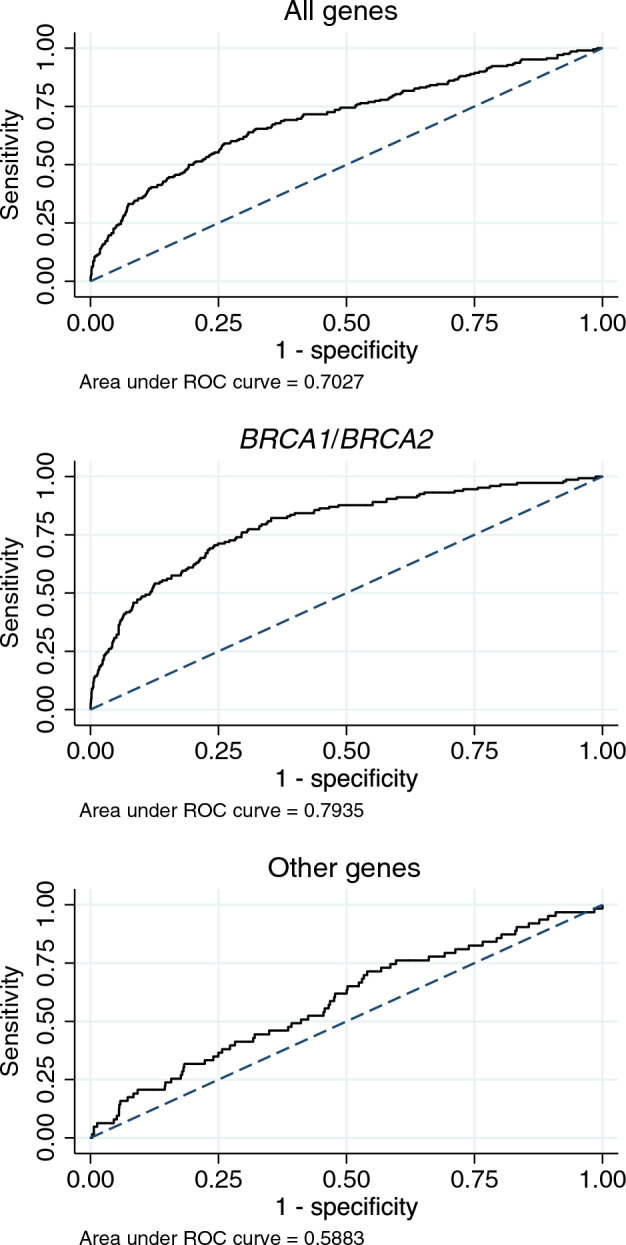
Receiver-operating characteristics for BOADICEA. The dashed line corresponds to a model with no ability to discriminate between carriers and non-carriers of PVs (area under ROC curve = 0.5). Top graph: all genes in the model. Middle graph: only *BRCA1* and *BRCA2*. Bottom graph: only the other genes in the model (*PALB2*, *CHEK2*, *ATM*, *BARD1*, *RAD51C* and *RAD51D*).

The AUC for all genes combined was slightly lower than the discrimination reported in validation studies of previous versions of BOADICEA, which only included *BRCA1* and *BRCA2* (e.g.^6^). However, the discrimination of BOADICEA was better for *BRCA1* and *BRCA2* (AUC 0.79; 95% CI 0.75–0.83) than for the other genes in the model (AUC 0.59; 95% CI 0.52–0.66) (Fig. 2). Likewise, an evaluation of the predictive performance of the model at individual cut-offs for the carrier probability showed higher sensitivity and specificity of predicting carriers of PVs in *BRCA1* and *BRCA2* compared to the other genes in the model (Table 3).

**Table 3:** Performance of BOADICEA at different carrier probability cut-offs for identifying carriers of a PV in *BRCA1*, *BRCA2*, *PALB2*, *CHEK2*, *ATM*, *BARD1*, *RAD51C* or *RAD51D*.

CP (%)	All genes		*BRCA1*/*BRCA2*		Other genes	
	Sensitivity	Specificity	Sensitivity	Specificity	Sensitivity	Specificity
≥ 0	1.00	0.000	1.00	0.000	1.00	0.000
≥ 1	1.00	0.001	1.00	0.001	1.00	0.001
≥ 2	0.98	0.072	0.98	0.071	0.97	0.068
≥ 3	0.92	0.19	0.95	0.19	0.87	0.18
≥ 4	0.83	0.37	0.87	0.36	0.73	0.35
≥ 5	0.75	0.51	0.83	0.51	0.56	0.49
≥ 10	0.50	0.80	0.61	0.80	0.25	0.77
≥ 15	0.38	0.89	0.47	0.89	0.21	0.86
≥ 20	0.31	0.93	0.39	0.93	0.14	0.91

*CP* Carrier probability for PVs (any gene).

The discrimination of BOADICEA did not vary much by the clinical data of the proband other than showing some improvement for clinical conditions more strongly associated with PVs in *BRCA1* than in the other genes, such as for ovarian cancer or ER and HER2 negative breast cancer (Supplementary Table S4).

### Individual genes

At the overall level, the model was well-calibrated for *BRCA2*, *CHEK2* and *ATM*. Significantly more PVs than expected were detected in *BRCA1* and less PVs than expected in *PALB2*. Notably, only four individuals were detected with PVs in *PALB2* compared to twenty expected carriers. The study was insufficiently powered to validate the predicted likelihood of carrying rare PVs in *RAD51C*, *RAD51D* and *BARD1* (Table 4).

**Table 4:** Calibration for the individual genes (N = 2033).

	O	E	O/E (95% CI)
*BRCA1*	85	48.7	1.75 (1.40–2.15)
*BRCA2*	61	57.3	1.06 (0.82–1.36)
*CHEK2*	37	34.3	1.08 (0.76–1.48)
*ATM*	15	14.7	1.02 (0.57–1.68)
*BARD1*	5	3.5	1.45 (0.47–3.37)
*PALB2*	4	20.2	0.20 (0.05–0.51)
*RAD51C*	1	4.3	0.23 (0.01–1.29)
*RAD51D*	1	4.3	0.23 (0.01–1.30)

*N* Total number of individuals, *O* Observed carriers of PVs, *E* Expected carriers of PVs.

There was statistical evidence of sub-optimal calibration for *BRCA1* (Hosmer–Lemeshow, P < 0.001) and *BRCA2* (P < 0.001), which both showed underestimation at low predicted likelihoods and overestimation at high predicted likelihoods similar to the results for the overall model and for these two genes combined. There was no evidence of mis-calibration for *CHEK2* (P = 0.98) or *ATM* (P = 0.70) with the caveat of fewer observations. In terms of discrimination, the AUCs were: *BRCA1* 0.83 (95% CI 0.78–0.87), *BRCA2* 0.76 (95% CI 0.68–0.83), *CHEK2* 0.68 (95% CI 0.60–0.76) and *ATM* 0.62 (95% CI 0.48–0.76). Calibration plots and ROC curves for these genes are provided in the supplement (Supplementary Fig. S2). For the remaining genes, observations were too few for further analysis.

### Missing information

The completeness of data was high for the probands. In most families, however, information was missing for at least one family member. The completeness of these data was lower in the Region of Southern Denmark (3.0%) than the Central Denmark Region (15.7%). In complete-case analysis of the 9.2% families with complete year of birth, age of death and age of cancer information on all family members, there was a trend towards underestimating the likelihood of carrying a PV compared to the study population as a whole. The same trend was found in complete-case analysis for year of birth information but not for age of death or age of cancer information isolated (Supplementary Table S5). As later discussed, these complete-case analyses could suggest that year of birth information was more likely to be complete in families with a detected PV, in which case family members would be offered genetic counselling and testing for the known PV. In a sensitivity analysis, we included the carrier status of the proband as a predictor in the imputations of missing data in their family. This did not significantly alter the calibration (O/E 1.11; 95% CI 0.97–1.26) or discrimination of the model (AUC 0.71; 95% CI 0.66–0.75).

## Discussion

Previous studies have demonstrated that BOADICEA in its earlier versions is highly accurate in predicting the likelihood of carrying PVs in *BRCA1* and *BRCA2*^5,6,9,17–19^. The discrimination of the model for *BRCA1* and *BRCA2* in the present evaluation was identical to that reported in the largest study to date^6^. The present study confirms that BOADICEA remains well-calibrated after the addition of *PALB2*, *CHEK2*, *ATM*, *BARD1*, *RAD51C* and *RAD51D*. It also establishes that the model remains significantly better than chance to discriminate between carriers and non-carriers of PVs in BC/OC susceptibility genes, such as *CHEK2*, in which PVs only add moderately to the risk of BC/OC. Notably, the discrimination of the model was significantly better for *BRCA1* and *BRCA2* than for the other genes in the model. The reduced ability to discriminate between carriers and non-carriers of PVs in these other genes did not necessarily reflect poor optimization of the model, however. A lower AUC would be expected given the lower penetrance of PVs in these genes compared to *BRCA1* and *BRCA2*. Since the performance of earlier versions of BOADICEA was broadly similar in European countries^9^, we expect the results of the present study to be likely applicable to other European populations although notable differences in population frequencies of some PVs could affect the results, such as for the frequent CHEK2 1100delC^20^. The accuracy of BOADICEA in non-European populations is less studied^21–23^; however, tailored models may be needed to improve the accuracy of the predicted likelihoods for individuals of non-European ancestry^24^.

For the development data, the first versions of BOADICEA were based on multiple UK family studies and meta-analysis of non-UK cohorts, including high-risk and population-based cases^25^. Later versions were made more specific to populations other than the UK, including the Danish, with population-specific cancer incidence rates^26^. Therefore, the populations for the development and validation data could be assumed to be broadly comparable, although a comparison of baseline characteristics between the different cohorts could not be made. At the overall level, there was a good correspondence between the observed and expected carriers of PVs across the different magnitudes of predicted likelihood. Yet in individuals with a predicted likelihood below five to ten percent, the observed frequency of PVs was higher than predicted by the model. Although the magnitude of difference is unlikely to be of significance in the genetic counselling, it may be of importance to consider when weighing the costs and benefits of offering genetic testing in the population according to a given threshold of predicted likelihood. With the decreasing cost of genetic testing, it could be argued that the usefulness of prediction models to assess the likelihood of carrying a PV will diminish over time; however, this is somewhat contradicted by the continued release of new prediction models^27^ alongside updates to the existing models such as BOADICEA^8^ and BRCAPRO^28^. Time will show the future respective needs for such prediction models in high- and low-income countries.

Whereas the calibration of BOADICEA was largely unaffected by the clinical data of the individual, the model tended to overestimate the likelihood of carrying PVs in individuals with a personal or family history of bilateral breast cancer and to underestimate this likelihood in individuals with a history of ovarian cancer. However, this did not affect the ability of the model to discriminate between carriers and non-carriers of PVs, which was good for both conditions. Moreover, the deviations of model calibration could be population-specific and would need to be confirmed in other populations. Of note, a new risk prediction model for ovarian cancer is already incorporated in CanRisk^29,30^, and a risk prediction model for contralateral breast cancer risk is underway.

Since gene panel testing is becoming increasingly widespread, what matters in the clinical setting is most likely the overall carrier probability; however, this could vary depending on local guidelines and the available resources. At the gene-level, BOADICEA slightly underestimated the likelihood of carrying PVs in *BRCA1* while it was well-calibrated for *BRCA2*. Underestimation of PVs in *BRCA1* has previously been reported in continental Europe as compared to the United Kingdom^5,6,18^, which may reflect differences in the population frequencies of PVs. Interestingly, we also found the model to overestimate the likelihood of PVs in *PALB2* in the present study population, which indicates that PVs represent a relatively rare cause of BC/OC susceptibility in Denmark. This suggests that the population frequencies of PVs in *PALB2* may be somewhat different in Denmark compared to those assumed in CanRisk. On the other hand, the cancer risks associated with PVs in *PALB2* are becoming more and more similar to those for *BRCA1* and *BRCA2*^1,31^. Thus, it is interesting that if the three genes were to be considered jointly the difference between the observed and expected count of PVs would decrease in the present study population. The model was well-calibrated for the remaining genes in which PVs are prevalent in individuals with BC/OC (*BRCA2*, *CHEK2*, *ATM*). For *BARD1*, *RAD51C* and *RAD51D*, PVs were too rare to validate the model with confidence, though the model appeared to be well-calibrated for *BARD1* as well.

The sensitivity of the genetic testing needed to be considered when evaluating the accuracy of BOADICEA, which predicts the likelihood of carrying a PV rather than the likelihood of detecting a PV. In parameterizing BOADICEA based on the estimated penetrance and population frequencies of PVs in previous studies, the developers of the model assumed the sensitivity of PV detection to be reduced in these studies due to unclassified missense PVs in *BRCA1* and *BRCA2* and the lack of CNV analysis for the remaining genes, for which the model only includes protein-truncating PVs^8^. The prevalence of unclassified missense PVs in *BRCA1* and *BRCA2* was estimated from the frequencies of rare variants in large case–control studies^1^, and these sensitivities have been included in CanRisk as the default option to estimate the likelihood of carrying a PV following a negative search for PVs. Because the present study included CNV analysis for all genes, the assumption of 100% sensitivity of genetic testing was in agreement with the parameterization of BOADICEA for *CHEK2*, *ATM*, *PALB2*, *BARD1*, *RAD51C* and *RAD51D*, whereas it would be reasonable in the evaluation of the model to assume a reduced sensitivity of genetic testing for *BRCA1* and *BRCA2* as unclassified missense PVs would also have been missed in the evaluation data set. Yet assuming reduced sensitivity of genetic testing for *BRCA1* and *BRCA2* corresponding to the estimated contribution of unclassified missense PVs in these genes did not significantly affect the observed calibration of the model indicating that the model will remain well-calibrated with improvements in classification of missense variants. The prevalence of non-coding PVs in *BRCA1* and *BRCA2* is unknown, but the loss of sensitivity due to failure to detect for example deep intronic variants is thought to be low^32^. It is also worth emphasizing, that we included both class 4 and class 5 variants in the category pathogenic. Although class 4 variants only have a 0.90–0.99 probability of being pathogenic^33^, these variants would also have been used for predictive testing in Denmark. Hence, the inclusion of these variants in the validation data set reflected standard clinical practice.

It is important to note that the ascertainment of the patients in the present study population may limit the generalizability of the findings. Because the patients were included from specialized clinical genetics departments, the results from this high-risk setting may not necessarily be all applicable to more population-based settings like primary care.

The study had other limitations. Like in other studies that make use of pedigree information collected in a clinical setting, information was missing for some pedigree members. Because of access to a national pathology database and the possibility to link the records of the proband using the central person register of Denmark, the completeness of data was very high for the patients who underwent genetic testing. In other cases, we would have to assume that the data was missing at random to allow imputation by univariable or multivariable regression models. A complete-case analysis suggested that some information was not missing completely at random. Hence, the observed calibration differed between the study population as a whole and individuals with complete information. Importantly, however, the calibration would only appear to differ with missing information about year of birth and not with missing information about age of death or age of cancer. Possibly, information about year of birth was more likely to be recorded in those families where a PV had been detected as additional family members would be offered predictive testing in these families. Worthy of notice, this does not necessarily imply a violation of the missing at random hypothesis that the missing information could be reliably predicted from the available data^34^. Given that we included the known years of births of family members in these imputations, the missing at random hypothesis appears plausible in the model, which was further supported by the fact that including the carrier status for PVs of the proband in the imputations of missing data did not significantly affect the results. Moreover, the year of birth has little effect on the estimated carrier probability compared to the age of death or age of cancer in BOADICEA. Altogether, since nearly complete information was available for the vast majority of individuals, any bias from violations of the missing at random assumption was most likely limited. Notably, even with some missing information the model could discriminate between carriers and non-carriers of PVs with no observable difference between the study population as a whole and the complete-case analysis. Data completeness also differed between the two departments, which likely reflected a difference in the clinical practice at the two departments for using the Danish civil registration system to complete information about year of birth and age of death of family members. This difference is unlikely to have affected the results of the present study. Another limitation of the study was the inability to adjust for family clustering. Some individuals originated from the same families. This could have led to a slight overestimation of the observed to expected ratio because searching for PVs in additional family members would cease when a PV was detected in one. However, we assumed any bias to be limited since testing for PVs would generally occur in parallel for all relevant family members. Furthermore, *BRCA1* and *BRCA2* were the only genes reported clinically and analyzed in real-time during most of the study period, whereas *PALB2*, *RAD51C* and *RAD51D* only entered at the very end of the study. Thus, PVs in genes other than *BRCA1* and *BRCA2* would not have been followed up at the clinical genetics departments. We cannot rule out that some pedigree members may have been tested for PVs before the study or in non-participating centers. It is possible that some pedigrees did not meet the outlined genetic testing criteria, as the decision to offer genetic testing could also be made by a clinical geneticist on other grounds, for example if the family history was deemed uninformative due to lack of contact. Unfortunately, we cannot know the extent of this because the family-based criteria meant that also individuals could be eligible for genetic testing by relation to another family member meeting the testing criteria. Also, we did not collect data on risk-reducing surgery in this cohort of families. The uptake of risk-reducing surgeries has previously been reported to be relatively high in families with hereditary BC/OC susceptibility in Denmark^35,36^. Yet the potential bias from previous testing or from following individuals no longer at risk for BC/OC is unlikely to be large because the majority of the patients were tested for PVs shortly after a diagnosis with cancer. Also, information about risk-reducing surgery in family members may not necessarily be available in the clinic. Due to few cases, the present study did not evaluate male breast cancer separately, but a validation study focused on this group of patients has previously demonstrated acceptable performance of the model for *BRCA1* and *BRCA2*^37^. Multifactorial risk factors and polygenic risk scores were not included in the present validation but could potentially improve the performance of the model. Finally, the usefulness of including cytokeratin 14 and cytokeratin 5/6 expression in the predictions to identify breast cancer of the basal-like phenotype will need to be evaluated where this information is available^38^.

Despite these limitations, the findings indicate that BOADICEA remains a valid decision-making aid for determining in a cost-effective manner which individuals to offer genetic testing for PVs in susceptibility genes for BC/OC. Furthermore, the study suggests that the model is well-calibrated for moderate penetrance genes such as *CHEK2* and *ATM*, which may have implications for the validity of cancer risk predictions. Future large-scale prospective studies of healthy carriers of PVs in these genes are needed to confirm the validity of the predicted cancer risk similar to previous validation studies for family history and polygenic risk score^39–42^. Yet it is interesting that a recent evaluation of BOADICEA version 6 suggests a high accuracy of the cancer risks associated with PVs jointly in the genes in the model^43^.

## Conclusions

This study confirms that BOADICEA remains a valid decision-making aid for determining which individuals to offer genetic testing for PVs in BC/OC susceptibility genes after the expansion of the model to include the effects of protein-truncating PVs in *CHEK2*, *ATM*, *PALB2*, *BARD1*, *RAD51C* and *RAD51D*. The calibration was most optimal at predicted carrier probabilities around ten to twenty percent, however, with evidence of underestimation at lower carrier probabilities and overestimation at higher carrier probabilities. Discrimination was significantly better for *BRCA1* and *BRCA2* than jointly for the other genes in the model. For the individual genes, the study confirms previous reports of underestimation for *BRCA1* in some populations, including Denmark. The significant overestimation for *PALB2* could be population-specific and needs to be confirmed in other populations. The study was insufficiently powered to validate the predictions for the rarest PVs, such as in *BARD1*, *RAD51C* and *RAD51D*. Altogether, questions remain about the clinical usefulness of predictions for these individual genes.

### Supplementary Information

Supplementary Information.

## Data Availability

The datasets generated and/or analysed during the current study are not publicly available due to them containing information that could compromise research participant privacy/consent but are available from the corresponding authors on reasonable request and in accordance with legal restrictions.

## Abbreviations

*ATM*: ATM serine/threonine kinase
AUC: Area under the curve
*BARD1*: BRCA1 associated RING domain 1
BOADICEA: The breast and ovarian analysis of disease incidence and carrier estimation algorithm
BC/OC: Breast cancer and/or ovarian cancer
*BRCA1*: BRCA1 DNA repair associated
*BRCA2*: BRCA2 DNA repair associated
*CHEK2*: Checkpoint kinase 2
CI: Confidence interval
CNV: Copy number variant
ER: Estrogen receptor
HER2: Human epidermal receptor 2
O/E: Observed-to-expected ratio
*PALB2*: Partner and localizer of BRCA2
PV: Pathogenic variant
*RAD51C*: RAD51 paralog C
*RAD51D*: RAD51 paralog D
