# Author Correction: Validation of the BOADICEA model for predicting the likelihood of carrying pathogenic variants in eight breast and ovarian cancer susceptibility genes

**DOI:** 10.1038/s41598-023-42606-z

**Published:** 2023-09-15

**Authors:** Nanna Bæk Møller, Desirée Sofie Boonen, Elisabeth Simone Feldner, Qin Hao, Martin Larsen, Anne‑Vibeke Lænkholm, Åke Borg, Anders Kvist, Therese Törngren, Uffe Birk Jensen, Susanne Eriksen Boonen, Mads Thomassen, Thorkild Terkelsen

**Affiliations:** 1 Department of Clinical Genetics, Aarhus University Hospital, Brendstrupgårdsvej 21, 8200 Aarhus N, Denmark; 2 Department of Clinical Genetics, Odense University Hospital, J. B. Winsløws Vej 4, 5000 Odense, Denmark; 3 Department of Surgical Pathology, Zealand University Hospital, Roskilde, Denmark; 4 Division of Oncology, Department of Clinical Sciences Lund, Lund University, Lund, Sweden

Correction to: *Scientific Reports* 10.1038/s41598-023-35755-8, published online 26 May 2023

The original version of this Article contained an error in Results section, under ‘Missing information’ subheading,

“This did not significantly alter the calibration (O/E 1.11; 95% CI 0.97–1.26) or discrimination of the model (AUC 0.71; 95% CI).”

now reads:

“This did not significantly alter the calibration (O/E 1.11; 95% CI 0.97–1.26) or discrimination of the model (AUC 0.71; 95% CI 0.66–0.75).”

Additionally, this Article contained errors in Table 3, where the sensitivity and specificity values for BRCA1/BRCA2 at CP ≥ 3% were incorrect. The correct and incorrect values appear below.

Incorrect:

**Table:** 

CP (%)	All genes		*BRCA1*/*BRCA2*		Other genes	
	Sensitivity	Specificity	Sensitivity	Specificity	Sensitivity	Specificity
≥ 0	1.00	0.000	1.00	0.000	1.00	0.000
≥ 1	1.00	0.001	1.00	0.001	1.00	0.001
≥ 2	0.98	0.072	0.98	0.071	0.97	0.068
≥ 3	0.92	0.19	0.87	0.36	0.87	0.18

Correct:

**Table:** 

CP (%)	All genes		*BRCA1*/*BRCA2*		Other genes	
	Sensitivity	Specificity	Sensitivity	Specificity	Sensitivity	Specificity
≥ 0	1.00	0.000	1.00	0.000	1.00	0.000
≥ 1	1.00	0.001	1.00	0.001	1.00	0.001
≥ 2	0.98	0.072	0.98	0.071	0.97	0.068
≥ 3	0.92	0.19	0.95	0.19	0.87	0.18

The original Article has been corrected.

