# Supplementary material for: Validation of the BOADICEA model for predicting the likelihood of carrying pathogenic variants in eight breast and ovarian cancer susceptibility genes
Source: Sci Rep. 2023 May 26;13:8536. doi: 10.1038/s41598-023-35755-8 (PMC10220031; doi:10.1038/s41598-023-35755-8)
Supplement: Supplementary file 1 — Supplementary Information. [file 41598_2023_35755_MOESM1_ESM.docx]

**Supplementary Methods**

*Data validation*

The year of birth and sex of the proband was known from the personal identification number. If this information differed from the pedigree database, the information from the personal identification number was assumed to be correct. Pedigree records with implausible values were manually inspected and typos corrected. If the correct values were not determined from the records, the information was handled as missing. Years of birth before 1850 were changed to 1850 to allow processing in BOADICEA (n = 4).

*Imputation of missing data*

Year of birth

Information about the year of birth was complete for the proband, but missing for some family members. It was imputed in the order starting from siblings, parents, siblings of parents to grandparents following these rules:

1. Siblings & siblings of parents: the mean year of birth in the generation of siblings
2. Parents & grandparents: the mean year of birth of their children subtracted by the cohort-specific mean difference in year of birth for the same type of family member

The year of birth was missing in 36% of all pedigree members.

Age of death

A missing age of death was imputed using a regression model with the covariates sex, year of birth and whether the individual had been affected by cancer (breast, bilateral breast, ovarian, pancreatic or prostate cancer). The year of birth was transformed using restricted cubic splines to model the non-linear relationship between the year of birth and the age of death. The age of death was missing in 37% of the pedigree members recorded as diseased.

Age of diagnosis

A missing age of diagnosis was imputed using a regression model with the covariates sex, year of birth and the age at latest follow-up. A missing age of diagnosis with bilateral breast cancer was imputed using a regression model with the covariates sex, year of birth, the age at follow-up and the age of the first breast cancer. The year of birth was transformed using restricted cubic splines to model the non-linear relationship between the year of birth and the age of diagnosis. The age of diagnosis was missing in the following proportion of the pedigree members with a recorded corresponding diagnosis: breast cancer 6.5%, bilateral breast cancer 2.5%, ovarian cancer 5.4%, prostate cancer 18.4% and pancreatic cancer 12.6%.

Outcome inclusion

A sensitivity analysis was performed where the carrier status of the proband for PVs was included as a covariate in the imputation of missing data for the family: For the year of birth, the cohort-specific mean difference in year of birth was stratified by the carrier status of the proband. For the age of death or age of diagnosis, the carrier status of the proband was included as a covariate in the regression models.

| **Supplementary Table S1.** Eligibility for genetic testing as recommended by The Danish Breast Cancer Cooperative Group during the study period (2013-2018) | |
| --- | --- |
| Criteria for genetic testing (any) | |
| - | Female diagnosed with breast cancer before the age of 40 |
| - | Female diagnosed with both breast and ovarian cancer |
| - | Female diagnosed with ER and HER2 negative or “basal-like” breast cancer before the age of 60* |
| - | Female diagnosed with ovarian cancer* |
| - | Two first degree relatives diagnosed with breast cancer before the age of 50 and/or ovarian cancer |
| - | Three first degree relatives diagnosed with breast cancer, at least one them before the age of 50 |
| - | Three first degree relatives diagnosed with breast cancer^*^ |
| - | Male diagnosed with breast cancer |
| - | Family with an identified pathogenic variant predisposing to breast and/or ovarian cancer |
| Eligible individuals: Affected family member, unaffected 1^st^ degree relative or 2^nd^ degree relative through a male | |
| *Starting from 2016.  Source: The Danish Breast Cancer Cooperative Group (www.dbcg.dk). | |

| **Supplementary Table S2**. Observed and expected numbers for the individual genes in the combined analysis for all genes. | | | | | | | | | | | | | | | | | | | | | | | |
| --- | --- | --- | --- | --- | --- | --- | --- | --- | --- | --- | --- | --- | --- | --- | --- | --- | --- | --- | --- | --- | --- | --- | --- |
|  | *BRCA1* | | *BRCA2* | | | *PALB2* | | | *CHEK2* | | | *ATM* | | | *BARD1* | | | *RAD51C* | | | *RAD51D* | | |
| CP (%) | O | E | | O | E | | O | E | | O | E | | O | E | | O | E | | O | E | | O | E |
| <5 | 17 | 3.2 | | 8 | 4.2 | | 1 | 3.3 | | 16 | 12.1 | | 7 | 5.3 | | 2 | 1.2 | | 1 | 1.5 | | 1 | 1.5 |
| 5-10 | 20 | 5.2 | | 12 | 9.8 | | 1 | 5.2 | | 12 | 11.4 | | 6 | 4.9 | | 0 | 1.1 | | 0 | 1.1 | | 0 | 1.1 |
| 10-15 | 13 | 5.0 | | 8 | 7.1 | | 0 | 3.2 | | 3 | 4.3 | | 0 | 1.8 | | 0 | 0.5 | | 0 | 0.5 | | 0 | 0.5 |
| 15-20 | 6 | 3.6 | | 5 | 5.5 | | 0 | 2.1 | | 1 | 2.3 | | 2 | 1.0 | | 1 | 0.2 | | 0 | 0.3 | | 0 | 0.3 |
| 20-30 | 14 | 6.0 | | 6 | 7.6 | | 1 | 2.3 | | 2 | 1.9 | | 0 | 0.8 | | 1 | 0.2 | | 0 | 0.4 | | 0 | 0.4 |
| 30-40 | 2 | 4.2 | | 6 | 6.5 | | 0 | 1.6 | | 1 | 1.2 | | 0 | 0.4 | | 1 | 0.1 | | 0 | 0.2 | | 0 | 0.2 |
| 40-50 | 2 | 4.6 | | 5 | 4.4 | | 1 | 0.7 | | 1 | 0.5 | | 0 | 0.2 | | 0 | 0.1 | | 0 | 0.1 | | 0 | 0.1 |
| ≥50 | 11 | 16.8 | | 11 | 12.1 | | 0 | 1.7 | | 1 | 0.8 | | 0 | 0.3 | | 0 | 0.1 | | 0 | 0.3 | | 0 | 0.2 |
| CP: Carrier probability for PVs (all genes); N: Total number of individuals; O: Observed carriers of PVs; E: Expected carriers of PVs | | | | | | | | | | | | | | | | | | | | | | | |

| **Supplementary Table S3**. Calibration for *BRCA1* and *BRCA2* versus *PALB2*, *CHEK2*, *ATM*, *BARD1*, *RAD51C* and *RAD51D* according to the predicted likelihoods | | | | | |
| --- | --- | --- | --- | --- | --- |
| CP (%) | | N | O | E | O/E (95% CI) |
| *BRCA1* and *BRCA2* | | | | | |
|  | <5 | 1,568 | 57 | 21.8 | 2.61 (1.99-3.37) |
|  | 5-10 | 215 | 20 | 15.2 | 1.31 (0.82-1.98) |
|  | 10-15 | 82 | 13 | 10.0 | 1.30 (0.72-2.10) |
|  | 15-20 | 44 | 14 | 7.6 | 1.84 (1.08-2.75) |
|  | 20-30 | 46 | 11 | 11.3 | 0.98 (0.51-1.58) |
|  | 30-40 | 25 | 7 | 8.6 | 0.82 (0.35-1.44) |
|  | 40-50 | 20 | 4 | 8.9 | 0.45 (0.13-0.98) |
|  | ≥50 | 33 | 20 | 22.6 | 0.89 (0.62-1.13 |
|  | Total | 2,033 | 146 | 106 | 1.38 (1.17-1.61) |
| *PALB2*, *CHEK2*, *ATM*, *BARD1*, *RAD51C* and *RAD51D* | | | | | |
|  | <5 | 1,577 | 43 | 47.0 | 0.91 (0.66-1.23) |
|  | 5-10 | 391 | 16 | 25.7 | 0.62 (0.36-1.00) |
|  | 10-15 | 51 | 3 | 6.1 | 0.49 (0.10-1.36) |
|  | ≥15 | 14 | 1 | 2.4 | 0.41 (0.01-1.94) |
|  | Total | 2,033 | 63 | 81.2 | 0.78 (0.60-0.99) |
| CP: Carrier probability for PVs (indicated genes); N: Total number of individuals; O: Observed carriers of PVs; E: Expected carriers of PVs; n.d.: Not determined. | | | | | |

| **Supplementary Table S4**. Calibration and discrimination according to the clinical information | | | | | | | |
| --- | --- | --- | --- | --- | --- | --- | --- |
|  |  |  | N | O | E | O/E (95% CI) | AUC (95% CI) |
| Sex | | | |  |  |  |  |
|  | Female | | 1,863 | 189 | 174 | 1.08 (0.94-1.24) | 0.71 (0.67-0.75) |
|  | Male | | 170 | 19 | 12.9 | 1.47 (0.91-2.23) | 0.64 (0.50-0.78) |
| Age | | | |  |  |  |  |
|  | <50 | | 816 | 90 | 77.4 | 1.16 (0.95-1.41) | 0.73 (0.68-0.79) |
|  | ≥50 | | 1,217 | 118 | 110 | 1.07 (0.90-1.27) | 0.68 (0.62-0.74) |
| Cancer diagnosis | | | |  |  |  |  |
|  | No cancer diagnosis | | 412 | 31 | 20.9 | 1.48 (1.02-2.07) | 0.65 (0.53-0.76) |
|  | Any cancer diagnosis | | 1,621 | 177 | 166 | 1.06 (0.92-1.22) | 0.71 (0.66-0.75) |
|  |  | Breast cancer | 1,334 | 137 | 146 | 0.94 (0.80-1.10) | 0.71 (0.66-0.76) |
|  |  | Bilateral breast cancer | 190 | 26 | 40.7 | 0.64 (0.43-0.91) | 0.73 (0.61-0.85) |
|  |  | Ovarian cancer | 301 | 49 | 31.0 | 1.58 (0.20-2.04) | 0.79 (0.72-0.86) |
|  |  | Prostate cancer | 32 | 3 | 2.8 | 1.07 (0.23-2.85) | 0.76 (0.31-1.00) |
|  |  | Pancreatic cancer | 18 | 3 | 2.0 | 1.48 (0.32-3.67) | 0.69 (0.08-1.00) |
| Breast cancer, receptor status | | | |  |  |  |  |
|  | ER+, HER2- | | 611 | 54 | 58.7 | 0.92 (0.70-1.18) | 0.70 (0.63-0.77) |
|  | ER-, HER2- | | 190 | 34 | 31.3 | 1.09 (0.77-1.46) | 0.81 (0.73-0.88) |
|  | ER+, HER2+ | | 131 | 12 | 12.1 | 0.99 (0.52-1.68) | 0.51 (0.29-0.72) |
|  | ER-, HER2+ | | 49 | 4 | 3.3 | 1.22 (0.34-2.92) | 0.64 (0.33-0.95) |
|  | Missing | | 353 | 33 | 40.2 | 0.82 (0.57-1.13) | 0.70 (0.60-0.81) |
| Family history (FH) | | | |  |  |  |  |
|  | No cancer FH | | 489 | 38 | 27.8 | 1.36 (0.98-1.85) | 0.63 (0.55-0.72) |
|  | Any cancer FH | | 1,544 | 170 | 159 | 1.07 (0.92-1.23) | 0.72 (0.67-0.76) |
|  |  | Breast cancer FH | 1,254 | 127 | 134 | 0.95 (0.80-1.11) | 0.71 (0.66-0.76) |
|  |  | Bilateral breast cancer FH | 158 | 22 | 34.7 | 0.63 (0.41-0.92) | 0.66 (0.52-0.80) |
|  |  | Ovarian cancer FH | 343 | 64 | 43.9 | 1.46 (1.15-1.81) | 0.72 (0.65-0.79) |
|  |  | Prostate cancer FH | 326 | 38 | 36.3 | 1.05 (0.75-1.40) | 0.70 (0.60-0.80) |
|  |  | Pancreatic cancer FH | 169 | 28 | 21.6 | 1.29 (0.88-1.80) | 0.77 (0.67-0.87) |
| N: Total number of individuals; O: Observed carriers of PVs; E: Expected carriers of PVs; AUC: Area under the receiver-operating characteristics curve | | | | | | | |

| **Supplementary Table S5**. Complete-case analysis | | | | | | |
| --- | --- | --- | --- | --- | --- | --- |
|  | | N | O | E | O/E (95% CI) | AUC (95% CI) |
| Study population | | 2,033 | 208 | 187 | 1.11 (0.97-1.26) | 0.70 (0.66-0.74) |
| Complete information* | |  |  |  |  |  |
|  | Proband | 2,024 | 207 | 186 | 1.11 (0.97-1.27) | 0.70 (0.66-0.74) |
|  | + 1^st^ degree relatives | 351 | 27 | 31.8 | 0.85 (0.57-1.21) | 0.68 (0.57-0.78) |
|  | + 2^nd^ degree relatives | 187 | 16 | 17.0 | 0.94 (0.55-1.49) | 0.70 (0.57-0.84) |
| Complete year of birth | |  |  |  |  |  |
|  | Proband | 2,033 | 208 | 187 | 1.11 (0.97-1.26) | 0.70 (0.66-0.74) |
|  | + 1^st^ degree relatives | 462 | 43 | 42.8 | 1.00 (0.74-1.33) | 0.67 (0.59-0.76) |
|  | + 2^nd^ degree relatives | 281 | 24 | 25.4 | 0.94 (0.61-1.38) | 0.70 (0.60-0.81) |
| Complete age of death | |  |  |  |  |  |
|  | Proband | 2,027 | 207 | 187 | 1.11 (0.97-1.26) | 0.70 (0.66-0.74) |
|  | + 1^st^ degree relatives | 662 | 72 | 64.6 | 1.11 (0.88-1.38) | 0.70 (0.64-0.76) |
|  | + 2^nd^ degree relatives | 526 | 57 | 51.7 | 1.10 (0.84-1.40) | 0.71 (0.65-0.78) |
| Complete age of cancer | |  |  |  |  |  |
|  | Proband | 2,030 | 208 | 186 | 1.12 (0.98-1.27) | 0.70 (0.66-0.74) |
|  | + 1^st^ degree relatives | 1,847 | 183 | 166 | 1.10 (0.95-1.26) | 0.70 (0.66-0.74) |
|  | + 2^nd^ degree relatives | 1,728 | 163 | 152 | 1.07 (0.92-1.24) | 0.69 (0.65-0.74) |
| *Excluding breast cancer pathology information (ER, PR, HER2, CK14, CK5/6) not included in imputations. N: Total number of individuals; O: Observed carriers of PVs; E: Expected carriers of PVs; AUC: Area under the receiver-operating characteristics curve | | | | | | |

**Supplementary Figure S1.** Calibration plots showing five quantiles of predicted likelihood. The spikes show the 95% CI for the observed likelihoods. Note the approximate intervals computed by ‘pmcalplot’ are slightly inaccurate at low observation numbers. The Hosmer-Lemeshow test assesses deviation from optimal calibration. Top graph: all genes in the model. Middle graph: only *BRCA1* and *BRCA2*. Bottom graph: only the other genes in the model (*PALB2*, *CHEK2*, *ATM*, *BARD1*, *RAD51C* and *RAD51D*).

**Supplementary Figure S2**. Calibration and discrimination for *BRCA1*, *BRCA2*, *CHEK2* and *ATM*. Left graphs: Calibration plots showing five quantiles of predicted likelihood. The spikes show the 95% CI for the observed likelihoods. Note the approximate intervals computed by ‘pmcalplot’ are slightly inaccurate at low observation numbers. The Hosmer-Lemeshow test assesses deviation from optimal calibration. Right graphs: Receiver-operating characteristics (ROC) curves for the same genes.
